# Light/Dark Cycle Lighting Influences Growth and Energy Use Efficiency of Hydroponic Lettuces in an LED Plant Factory

**DOI:** 10.3390/biology14050571

**Published:** 2025-05-20

**Authors:** Wen Li, Luming Zhong, Xiang Ji, Jun Wang, Dongxian He

**Affiliations:** 1 College of Water Resources and Civil Engineering, China Agricultural University, Beijing 100083, China; li.wen@cau.edu.cn (W.L.); b20223090736@cau.edu.cn (L.Z.); jx@cau.edu.cn (X.J.); 2 Key Laboratory of Agricultural Engineering in Structure and Environment, Ministry of Agriculture and Rural Affair, Beijing 100083, China; 3 Institute of Vegetables and Flowers, Chinese Academy of Agricultural Sciences, Beijing 100081, China

**Keywords:** circadian rhythm, photosynthetic rate, biomass, cultivar

## Abstract

The alterable light/dark cycle in a plant factory with artificial lighting eliminates the traditional concept of day and night in nature. Adjusting the light/dark cycle to closely align with the inherent circadian rhythm of plants can enhance biomass accumulation. In this study, we examined the effects of different light/dark cycles on the photosynthetic performance, growth, and energy use efficiency of two hydroponic lettuce cultivars (*Lactuca sativa* L. cv. ‘Frillice’ and ‘Crunchy’). The lettuces were subjected to four light/dark cycle treatments—16 h light/8 h dark (L_16_D_8_, as control), 12 h light/6 h dark (L_12_D_6_), 8 h light/4 h dark (L_8_D_4_), and 4 h light/2 h dark (L_4_D_2_), all under LED lamps with white combined red chips at the same light intensity of 250 μmol m^−2^ s^−1^. Photosynthetic performance and growth index were measured during the slow and rapid growth stages, corresponding to days 9 and 21 after transplanting, respectively. For Frillice, L_12_D_6_ achieved the highest shoot dry weight and light and electricity energy use efficiencies on days 9 and 21 after transplanting, primarily due to the largest leaf area, leaf number, and net photosynthetic rate. For Crunchy, L_12_D_6_ and L_8_D_4_ increased shoot fresh and dry weights due to larger leaf area and leaf number on day 9 after transplanting compared with L_16_D_8_. Subsequently, the lettuces in L_16_D_8_ exhibited a rapid increase in leaf area and leaf number, along with a high net photosynthetic rate during the rapid growth stage, resulting in fast shoot biomass accumulation. There were no significant differences in the shoot dry weight and energy use efficiency between L_16_D_8_ and L_12_D_6_ on day 21 after transplanting. Two lettuce cultivars in L_16_D_8_ both exhibited the highest water use efficiency on day 21 after transplanting. In conclusion, the light/dark cycle lighting can alter lettuce biomass accumulation by modifying plant morphology and leaf net photosynthetic rate. Additionally, the physiological response to the light/dark cycle was cultivar-dependent. Our findings provide valuable insights for optimizing hydroponic lettuce production to achieve high yield in LED plant factories.

## Simple Summary

Adjusting the light/dark cycle to synchronize closely with the circadian rhythm could enhance plant growth. Hence, the effects of a light/dark cycle on two hydroponic lettuce cultivars (*Lactuca sativa* L. cv. ‘Frillice’ and ‘Crunchy’) were investigated at different growth stages in an LED plant factory. Lettuces were subjected to 16 h light/8 h dark (L_16_D_8_, as control), L_12_D_6_, L_8_D_4_, and L_4_D_2_. For Frillice, only L_12_D_6_ significantly enhanced the shoot dry weight at both stages and energy (light and electric) use efficiency, resulting from the increased leaf area, leaf number, and photosynthetic rate. However, for Crunchy, growth advantage under L_12_D_6_ at the slow growth stage vanished at the rapid growth stage. Only L_4_D_2_ reduced the biomass accumulation at the rapid growth stage compared with L_16_D_8_. In conclusion, the light/dark cycle influenced lettuce growth by altering morphology and photosynthesis. These findings offer guidance for the commercial production of different lettuce cultivars in the plant factory.

## 1. Introduction

Increasing population and unpredictable climate disasters have resulted in growing food scarcity [1,2,3]. Plant factories with artificial lighting, as a form of environmentally controlled agricultural cultivation, could partly alleviate the anabatic pressure. At present, popularized plant factories generally install LED lamps due to their high luminous efficacy and ability to gradually reduce production cost [4,5]. The cultivation of leaf vegetables is highly valued in commercial plant factories because of their fast growth and low requirement for light intensity and space. Lettuce (*Lactuca sativa* L.), rich in vitamins and minerals, has become the fifth most popular crop, just behind corn, potato, rice, and tomato [6,7]. In addition, it is also a model plant for photobiology research [8].

The light/dark cycle is characterized by the length of the cycle (period) and the ratio of light time to dark time [9]. In nature, the circadian clock generates a circadian rhythm with a period of approximately 24 h, which is equal to a rotation of the Earth. Breaking the traditional concept of 24 h is possible in an LED plant factory by powering the lighting on or off. The light/dark cycle period will be unlimited, and can be below or above 24 h. Moreover, light/dark ratio on a second-scale or hour-scale could also be freely regulated to achieve high productivity. It has been reported that the light/dark cycle could influence plant growth at the morphological, physiological, biochemical, and molecular levels [10,11,12,13,14].

In lettuce, different light/dark cycles result in distinct plant growth performance, depending on cultivars. Under an unchanged daily light integral, increased light/dark cycle periods with equivalent light time and dark time could enhance biomass, leaf number, leaf area, and photosynthetic rate in lettuce (*L. var*. *Capitata* L. ‘Adriana’ and ‘Greenwave’) [15,16,17]. However, lettuces (*L.* ‘Hongyeom Jeockchukmyeon’ and ‘Cos’) exhibited no statistically significant difference in shoot fresh or dry weight under different light/dark cycles [18]. Additionally, some lettuce cultivars did not show biomass accumulation along a single direction with an extended or shortened light/dark cycle period. For example, the shoot weight of lettuce (*L. crispa* ‘Green Oak Leaf’) increased under light/dark cycles of 8/4, 4/2, and 2/1, whereas it decreased under a light/dark cycle of 6/3 together with 4/2, compared with a light/dark cycle of 16/8 [10]. Butter lettuce (*L.* ‘Flandria’) subjected to light/dark cycles of 16/8, 24/12, 48/24, 96/48, and 120/60 exhibited the highest shoot fresh weight under a light/dark cycle of 24/12 [9]. Romaine lettuce (*L.* ‘Ideal-205′) grown under light/dark cycles of 16/8 and 8/4 exhibited higher dry weight than that under a light/dark cycle of 12/6 [11]. In addition to the different cultivars, another reason for the different results was the experimental design, e.g., growth light intensity and light quality. In addition, the synchronization of the endogenous physiology and metabolism with the external light/dark cycle could contribute to conferring a substantial photosynthetic advantage, resulting in potentially high yield [19].

Resource use efficiencies are commonly used to evaluate the performance of plant factories [20]. Among indicators, light energy use efficiency (LUE) and electric energy use efficiency (EUE) are mainly considered due to their generous use of lighting. It has been reported that LUE ranges from 3.2% to 4.3% in the plant factory; meanwhile, EUE is approximately 0.7% based on the conversion coefficient from electric energy to light energy [20]. Under the same light sources, the optimization of light strategy is a primary approach to maximizing biomass accumulation. Together with the development of the luminous efficacy of LED lamps, an optimal light/dark cycle could improve LUE and EUE, which reach up to more than 5.0% and 1.5%, respectively [9]. The promotion is attributed to enhanced photosynthetic performance and adjusted leaf shape and leaf angle [9,15], leading to maximized light interception [15].

Previous studies mainly focused on the effect of the light/dark cycle on the harvested lettuce. Lettuce growth response to light/dark cycle at the different growth stages has rarely been considered. We hypothesized that lettuce responds to light/dark cycle in a cultivar-dependent manner. Therefore, the impacts of different light/dark cycles on the photosynthetic performance, growth, and energy use efficiency of two typical hydroponic lettuce cultivars at the slow and rapid growth stages were investigated. Our results provide new insights for optimizing hydroponic lettuce production to achieve high yield and energy utilization in an LED plant factory. Further, an optimized light environment strategy would help guide commercial horticultural practice and enhance the sustainable development of plant factories.

## 2. Materials and Methods

### 2.1. Seedling Conditions and Plant Material

The experiment was conducted in a plant factory laboratory at China Agricultural University (40°0′ N, 116°21′ E). For hydroponic lettuce seedlings, two individual shelves (1.25 m length × 0.60 m width × 2.10 m height) were laid out in the plant factory. Each shelf consisted of four layers with 0.38 m height per layer. Each layer was installed with LED lamps (WR-16W, Beijing Lighting Valley Technology Co., Ltd., Beijing, China), which were composed of white combined red chips. To keep light intensity uniform on the same plane, LED lamps were installed with irregular horizontal spacing. Moreover, the shelves were surrounded by reflective film. Each layer contained a cultivation bed (1.20 m length × 0.60 m width × 0.07 m height) made of acrylonitrile butadiene styrene.

A fully soaked whole sponge was placed in a plastic tray (33.5 cm length × 27.0 cm width × 5.0 cm height). Lettuce (*L.* cv. ‘Frillice’ and ‘Crunchy’) seeds were directly sown in the middle holes of the polyurethane sponge blocks (23 mm length × 23 mm width × 23 mm height, Shengjie Sponge Products Factory, Jiangmen, China), which were discarded after use. Then, the trays were covered with plastic film to keep the moisture inside. After 2 days of darkness, sowed lettuces were placed under 200 μmol m^−2^ s^−1^ photosynthetic photon flux density emitted by LED lamps with red/blue ratio of 3:1 for 21 days. Light intensity was measured at the distance of 15 cm from the bottom of the lamp using a fiber optical spectrometer (AvaFiled-2, Avantes Inc., Apeldoorn, The Netherlands).

Photoperiod, daytime air temperature, relative humidity, and CO_2_ concentration in the seedling environment were 16 h d^−1^, 21 °C, 70%, and 800 μmol mol^−1^, respectively. Nighttime air temperature, relative humidity, and CO_2_ concentration were 18 °C, 65%, and not controlled, respectively. The seedling density was 118 plants m^−2^. The modified Yamazaki lettuce nutrient solution recipe (electrical conductivity, 1.6 mS cm^−1^; pH, 6.0) was adopted. The nutrient solution composition (mg L^−1^) was as follows: 5Ca(NO_3_)_2_·NH_4_NO_3_·10H_2_O 432.2, KNO_3_ 808.0, NH_4_H_2_PO_4_ 152.0, MgSO_4_·H_2_O 246.0, EDTA-Fe-13 30.8, H_3_BO_3_ 5.72, MnSO_4_·H_2_O 3.22, ZnSO_4_·H_2_O 0.28, (NH_4_)_6_Mo_7_O_24_·4H_2_O 0.04, CuSO_4_·5H_2_O 0.16. Once the first true leaves grew out, the lettuces were irrigated with 1/2 strength nutrient solution. When the second true leaves grew out, full-strength nutrient solution was adopted.

### 2.2. Light/Dark Cycle Treatments

Seedlings with more than three true leaves (approximately 1.0 g plant^−1^) were selected and transplanted to eight cultivation beds with a density of 36 plants m^−2^. Two lettuce cultivars were both subjected to four light/dark cycle treatments: 16 h light/8 h dark (L_16_D_8_, as control), 12 h light/6 h dark (L_12_D_6_), 8 h light/4 h dark (L_8_D_4_), and 4 h light/2 h dark (L_4_D_2_). Four light treatments for each cultivar were distributed into four layers of the same shelf. The light intensity, air temperature, relative humidity, and CO_2_ concentration were 250 μmol m^−2^ s^−1^, 20 °C, 70%, and 800 μmol mol^−1^, respectively. The light integral over 3 days of all treatments was maintained at the same value of 43.2 mol m^−2^. The total photons emitted by LED lamps for each treatment were uniform. The light/dark cycles in the cultivation period of 21 days for L_16_D_8_, L_12_D_6_, L_8_D_4_, and L_4_D_2_ were 21, 28, 42, and 84, respectively (Table 1). After transplanting, Frillice and Crunchy exhibited notable discrepancies in shoot fresh weight on the day-scale (Figure S1). The lettuce growth process could be divided into slow and rapid growth stages. Unless specifically stated, the samples were collected on days 9 and 21 after transplanting, which corresponded to slow and rapid stages, respectively.

Due to high evapotranspiration during the cultivation period, a dehumidifier (DYD138, Qingdao Deyoujiang Electrical Equipment Manufacturing Co., Ltd., Qingdao, China) was installed to regulate humidity at the setting value. The nutrient solution was automatically regulated using a fertilizer to maintain electrical conductivity and pH at the setting values of 1.6 mS cm^−1^ and 6.0, respectively.

### 2.3. Photosynthetic Parameters and Chlorophyll Fluorescence

The photosynthetic gas exchange of the third fully expanded leaf was determined using portable photosynthesis equipment with a leaf chamber of 6400-02B (LI-6400XT, LI-COR Inc., Lincoln, NE, USA) on days 9 and 21 after transplanting [21]. Light intensity, leaf temperature, CO_2_ concentration, and flow rate in the cuvette were 250 μmol m^−2^ s^−1^, 20 °C, 800 μmol mol^−1^, and 400 μmol s^−1^, respectively. Water use efficiency was calculated as the ratio of net photosynthetic rate to transpiration rate.

The chlorophyll fluorescence of the same sampled leaf was determined using Dual-PAM-100 (Heinz Walz GmbH, Effeltrich, Germany). The leaves were dark-adapted for 30 min using leaf clamp, then minimum fluorescence (F_o_) and maximum fluorescence (F_m_) were measured. Dark-adapted maximal Photosystem II (PSII) efficiency (F_v_/F_m_) was calculated as F_v_/F_m_ = 1 − F_o/_F_m_. Subsequently, the leaf was exposed to actinic light of 540 μmol m^−2^ s^−1^ to achieve fluorescence yield (F_s_) and maximal fluorescence of light-adapted leaf (F_m_′). PSII photochemical efficiency (ΦPSII) and electron transport rate through PSII (ETR) were calculated according to the equations [22] ΦPSII = (F_m_′ − F_s_)/F_m_′ and ETR = ΦPSII·α·PPFD·0.5, where α is leaf absorptance, which is typically assumed to be 0.84. The factor 0.5 assumes that absorbed quanta are used equally to excite photosystem I and PSII. Four plants were sampled for the measurement of photosynthetic parameters and chlorophyll fluorescence.

### 2.4. Growth Parameters

Five plants were randomly sampled to determine leaf number and shoot fresh weight. For the measurement of leaf area, the leaves were entirely flattened on a background panel with a quadrate mark (1 cm × 1 cm), then scanned using a CanoScan LiDE400 (Canon Inc., Tokyo, Japan). The pixel areas of the quadrate mark and leaves in the images were both calculated using Adobe Photoshop 2022 (Adobe Inc Co., Ltd, San Jose, CA, USA), then the leaf area (cm^2^) was acquired via the conversion of pixel area (leaves pixel area/quadrate mark pixel area). Plants were placed into an oven at 75 °C for 72 h until constant values became available for the determination of shoot dry weight. Growth rate was calculated according to the following equation:

Growth rate = ΔDW/interval days(1)

Here, ΔDW (g plant^−1^) was the increment of shoot dry weight per plant.

### 2.5. Photon Yield and Energy Use Efficiency

Photon yield (g mol^−1^) represents the produced fresh weight of the available part of the plant derived from photons received within the waveband of 300–800 nm. The equation is as follows [23]:

Photon yield = ΔFW × density/(daily light integral × days)(2)

Here, ΔFW (g plant^−1^) was the increment of shoot fresh weight per plant. The daily light integral (mol m^−2^ d^−1^) was the cumulative photosynthetic photon flux density per day at the plant canopy. Days were counted from transplanting to harvest, for a total of 21 days in this study.

Light energy use efficiency and electric energy use efficiency represent the chemical energy produced by the available part of the plant derived from the received radiation energy and consumed electric energy, respectively. The equations are as follows [20]:

Light energy use efficiency = f × ΔDW/1000 × density/R(3)

Electric energy use efficiency = f × ΔDW/1000 × density/E(4)

Here, f (MJ kg^−1^) is the conversion factor from dry mass per kilogram to chemical energy (approximately 20); R (MJ m^−2^) is the received radiation energy within the range of 300–800 nm at the plant canopy. E (MJ m^−2^) is the consumed electric energy.

### 2.6. Statistical Analysis

Data are presented as means ± standard deviation (±SD) for each treatment. The data were first tested for normality (Shapiro–Wilk test) and homogeneity of variances (Levene test) using SAS software ver. 9.1 (Statistical Analysis System Institute Inc., Cary, NC, USA). We found that all data fit the homogeneity of variance (*p* > 0.05). Subsequently, one-way analysis of variance (ANOVA) was performed followed by Duncan’s multiple range test at a 95% confidence level. Figures were drawn using GraphPad Prism ver. 10 (GraphPad Software Inc., San Diego, CA, USA).

## 3. Results

**Figure 1:**
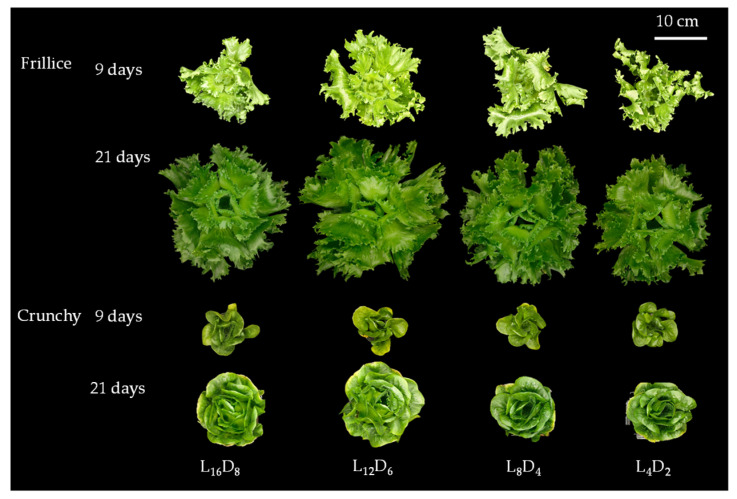
Morphology of lettuces (cv. Frillice and Crunchy) in response to different light/dark cycles on days 9 and 21 after transplanting in the LED plant factory. L_16_D_8_, 16 h light/8 h dark; L_12_D_6_, 12 h light/6 h dark; L_8_D_4_, 8 h light/4 h dark; L_4_D_2_, 4 h light/2 h dark.

We investigated leaf number, leaf area, photosynthetic parameters, and chlorophyll fluorescence to evaluate the impacts of light/dark cycle on lettuce growth. Typical morphological differences were found in Frillice and Crunchy, related to crisp and butterhead lettuce, respectively (Figure 1). Different lettuce cultivars exhibited distinct growth parameters in response to light/dark cycles during both slow and rapid growth stages.

### 3.1. Leaf Number and Leaf Area

**Figure 2:**
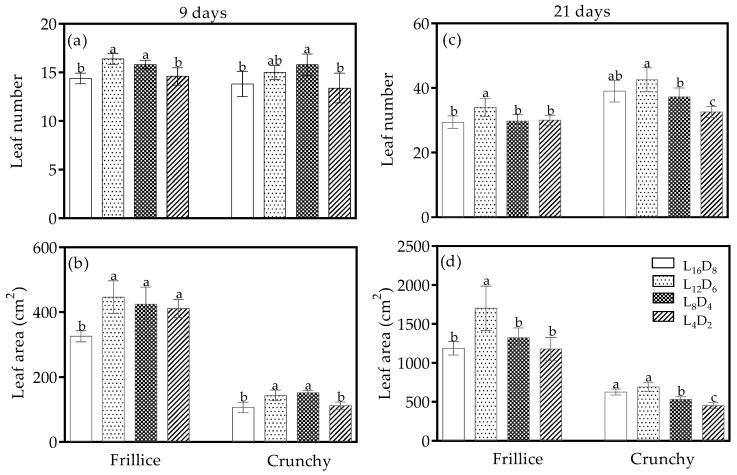
Leaf number (**a**,**c**) and leaf area (**b**,**d**) of lettuces (cv. Frillice and Crunchy) in response to different light/dark cycles on days 9 and 21 after transplanting. Different letters for the same parameter indicate significant differences at the 5% level, according to Duncan’s multiple range test (*n* = 5).

Leaf morphology was affected by light/dark cycle. On day 9 after transplanting, in Frillice, the leaf number and leaf area were 9.7–13.9% and 30.2–36.9% higher under L_12_D_6_ and L_8_D_4_ than under L_16_D_8_ (Figure 2a,b). Additionally, lettuce under L_4_D_2_ also exhibited a 26.2% increase in leaf area. In Crunchy, leaf number significantly increased only under L_8_D_4_ compared with L_16_D_8_, while leaf area increased by 35.4–43.1% under L_12_D_6_ and L_8_D_4_ (Figure 2a,b). However, there was no significant difference in leaf number and leaf area between L_16_D_8_ and L_4_D_2_.

On day 21 after transplanting, in Frillice, both leaf number and leaf area were 15.6% and 43.2% higher under L_12_D_6_ than under L_16_D_8_; in Crunchy, while no significant differences were observed in leaf number or leaf area between L_16_D_8_ and L_12_D_6_ (Figure 2c,d). However, L_8_D_4_ and L_4_D_2_ caused reductions of 4.6–16.4% in leaf number and 15.2–27.8% in leaf area compared with the control.

### 3.2. Photosynthetic Parameters

**Figure 3:**
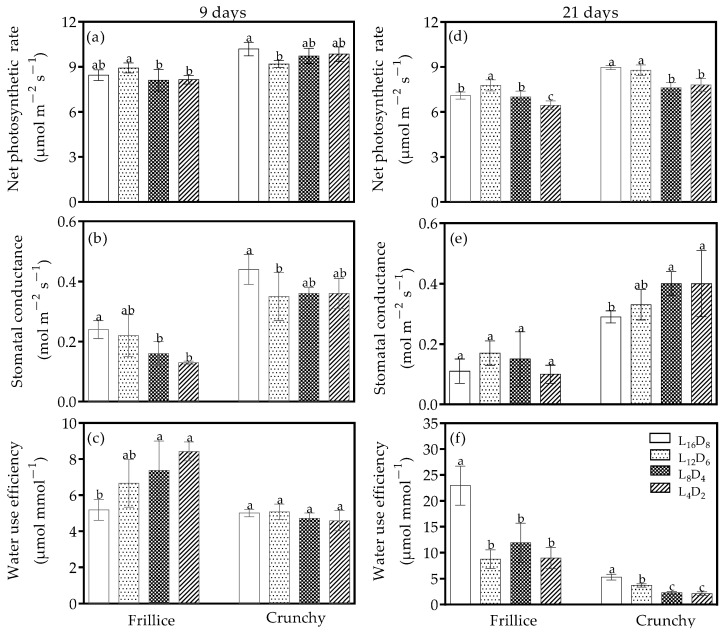
Net photosynthetic rate (**a**,**d**), stomatal conductance (**b**,**e**), and water use efficiency (**c**,**f**) of lettuces (cv. Frillice and Crunchy) in response to different light/dark cycles on days 9 and 21 after transplanting. Different letters for the same parameter indicate significant differences at the 5% level, according to Duncan’s multiple range test (*n* = 5).

Photosynthetic parameters were altered following different light/dark cycles and significant treatment effects were observed on two lettuce cultivars. On day 9 after transplanting, in Frillice, net photosynthetic rate, stomatal conductance, and water use efficiency were all unaffected by L_12_D_6_ compared with its respective L_16_D_8_ (Figure 3a–c). Net photosynthetic rate and stomatal conductance were 3.6–4.1% and 34.9–48.1% lower under L_8_D_4_ and L_4_D_2_ than under L_16_D_8_, respectively (Figure 3a,b). Conversely, water use efficiency increased by 42.1–62.4% under L_8_D_4_ and L_4_D_2_ compared with its control (Figure 3c). In Crunchy, reductions in net photosynthetic rate and stomatal conductance only under L_12_D_6_ were observed (Figure 3a,b), while water use efficiency remained unchanged across all light/dark cycles compared with its respective control (Figure 3c).

On day 21 after transplanting, in Frillice, net photosynthetic rate increased under L_12_D_6_ and decreased under L_4_D_2_ (Figure 3d), while stomatal conductance did not change across all light/dark cycles (Figure 3e). In Crunchy, net photosynthetic rate and stomatal conductance were unaffected by L_12_D_6_ compared with its respective L_16_D_8_ (Figure 3d,f). However, net photosynthetic rate declined by 12.9–15.0% under L_8_D_4_ and L_4_D_2_ compared with its control (Figure 3d), while stomatal conductance increased by 36.4–37.7% under L_8_D_4_ and L_4_D_2_ (Figure 3e). In both Frillice and Crunchy, altered light/dark cycles significantly reduced water use efficiency within the cultivar (Figure 3f).

### 3.3. Chlorophyll Fluorescence

**Figure 4:**
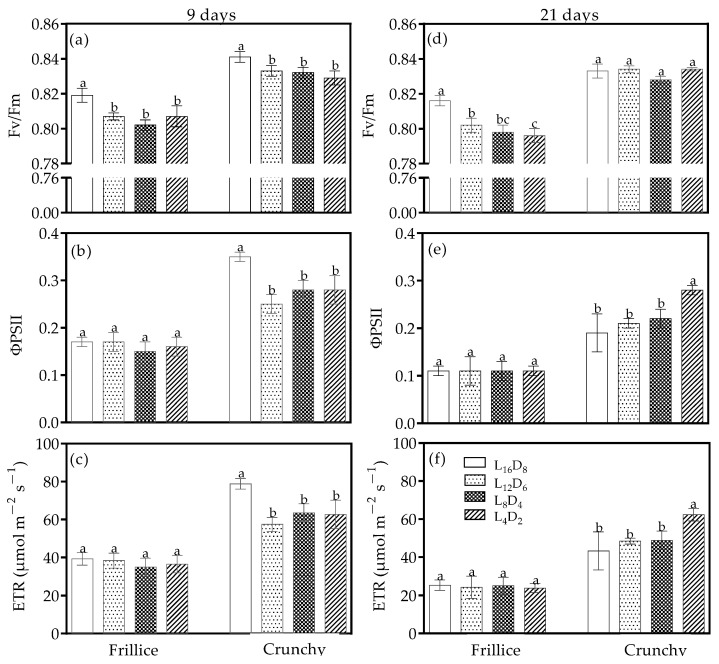
Dark-adapted maximal Photosystem II (PSII) efficiency (F_v_/F_m_) (**a**,**d**), PSII photochemical efficiency (ΦPSII) (**b**,**e**), and electron transport rate through PSII (ETR) (**c**,**f**) of lettuces (cv. Frillice and Crunchy) in response to different light/dark cycles on days 9 and 21 after transplanting. Different letters for the same parameter indicate significant differences at the 5% level, according to Duncan’s multiple range test (*n* = 4).

To investigate whether light/dark cycles induced distress or not in lettuce, F_v_/F_m_ was measured. F_v_/F_m_ was slightly lower for both cultivars under all altered light/dark cycles than that under their respective L_16_D_8_ on day 9 after transplanting, with values ranging from 0.80 to 0.84 (Figure 4a). ΦPSII was affected by light/dark cycle depending on cultivars. In Frillice, ΦPSII and ETR were unaffected by light/dark cycles (Figure 4b,c). Similar results were found on day 21 after transplanting (Figure 4e,f). However, ΦPSII and ETR were 19.3–27.2% and 19.3–27.1% lower under all altered light/dark cycles in Crunchy than that under their respective L_16_D_8_, respectively (Figure 4b,c). On day 21 after transplanting, only L_4_D_2_ induced 44.2% and 44.1% increases in ΦPSII and ETR for Crunchy compared with its control (Figure 4e,f). On day 21 after transplanting, all altered light/dark cycles in Frillice reduced F_v_/F_m_, but no significant difference was observed in Crunchy (Figure 4d).

### 3.4. Shoot Weight and Growth Rate

**Figure 5:**
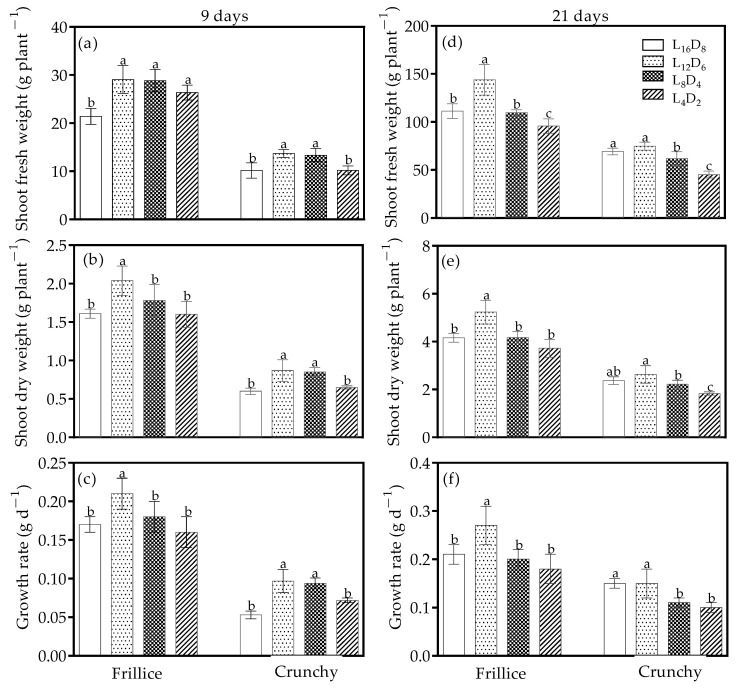
Shoot fresh weight (**a**,**d**), shoot dry weight (**b**,**e**), and growth rate (**c**,**f**) of lettuces (cv. Frillice and Crunchy) in response to different light/dark cycles on days 9 and 21 after transplanting. Different letters for the same parameter indicate significant differences at the 5% level, according to Duncan’s multiple range test (*n* = 5).

Biomass was affected by the light/dark cycle within the cultivar. On day 9 after planting, in Frillice, shoot fresh weight increased by 23.1–35.7% under all altered light/dark cycles compared with its respective L_16_D_8_ (Figure 5a). Only L_12_D_6_ caused an increase in shoot dry weight and growth rate compared with its control (Figure 5b,c). In Crunchy, shoot fresh weight, shoot dry weight, and growth rate were 31.4–34.6%, 41.0–45.6%, and 76.2–81.9% higher under L_12_D_6_ and L_8_D_4_ than that under L_16_D_8_, respectively (Figure 5a–c). On day 21 after transplanting, in Frillice, shoot fresh weight, shoot dry weight, and growth rate were only increased by L_12_D_6_, with 29.4%, 25.8%, and 25.0% increases compared with its respective control (Figure 5d–f). However, all the above indexes were unaffected by L_12_D_6_ in Crunchy. A reduction in shoot fresh weight under L_4_D_2_ in Frillice was observed, whereas shoot dry weight and growth rate were not affected (Figure 5d–f). In Crunchy, shoot fresh weight, shoot dry weight, and growth rate were 10.1–34.5%, 6.6–23.0%, and 22.7–33.6% lower under L_8_D_4_ and L_4_D_2_ than under L_16_D_8_ (Figure 5d–f). Besides this, L_4_D_2_ aggravated the adverse effects on shoot weight and growth rate compared with L_8_D_4_.

### 3.5. Photon Yield, Light Energy Use Efficiency, and Electric Energy Use Efficiency

**Figure 6:**
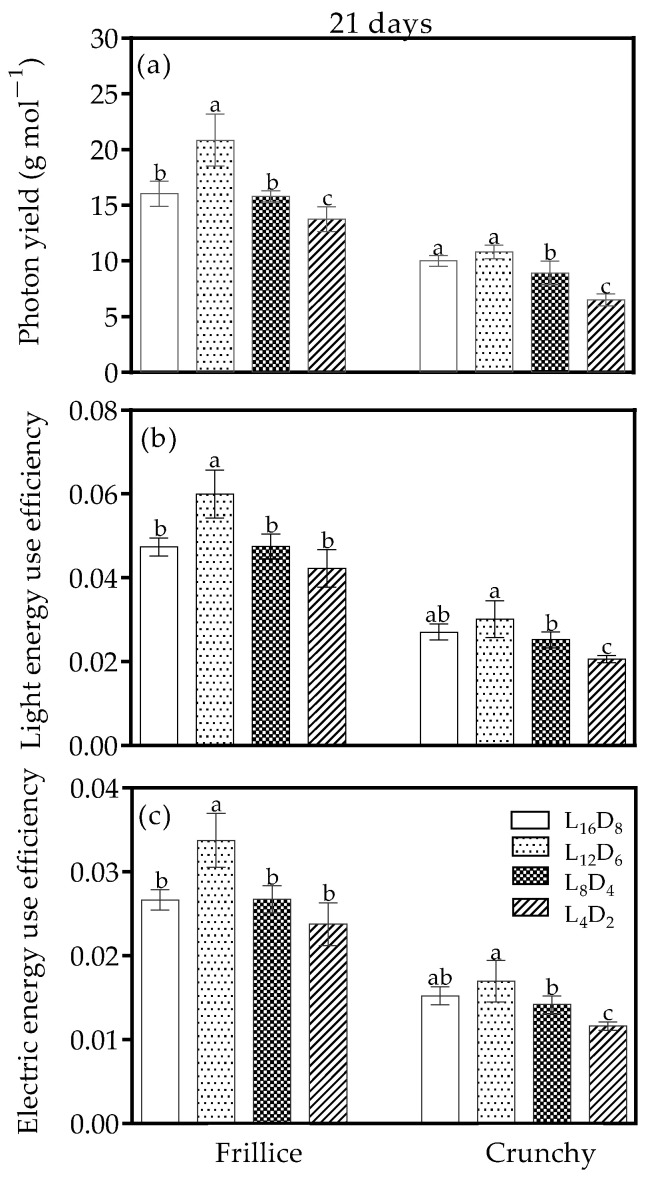
Photon yield (**a**), light energy use efficiency (**b**), and electric energy use efficiency (**c**) of lettuces (cv. Frillice and Crunchy) in response to different light/dark cycles on day 21 after transplanting. Different letters for the same parameter indicate significant differences at the 5% level, according to Duncan’s multiple range test (*n* = 5).

The different light/dark cycles resulted in distinct photon yields and energy use efficiencies at harvest. In Frillice, only L_12_D_6_ caused increases in photon yield, light energy use efficiency, and electric energy use efficiency, with increases of 29.9%, 26.6%, and 26.6%, respectively (Figure 6a–c). However, all above indexes were unaffected by L_12_D_6_ in Crunchy compared with its respective L_16_D_8_. L_4_D_2_ reduced the photon yield by 14.2–35.0% in both Frillice and Crunchy compared with their respective controls (Figure 6a). Nevertheless, L_8_D_4_ had no influence on light energy use efficiency and electric energy use efficiency in either Frillice or Crunchy (Figure 6b,c). L_4_D_2_ led to unchanged light energy use efficiency and electric energy use efficiency in Frillice, whereas an obvious reduction was observed in Crunchy.

## 4. Discussion

### 4.1. Lettuce Response to Light/Dark Cycle Was Cultivar-Dependent

The circadian clock is composed of three key components: the input pathway, the endogenous oscillator, and the output pathway. The endogenous oscillator generates the circadian rhythm, which synchronizes with external environmental cues, such as light, temperature and phytohormones, to regulate various physiological processes [24]. Among these cues, the light/dark signal is the most powerful zeitgeber, capable of entraining cellular oscillators and resetting them to achieve complete synchronization [25]. Aligning the endogenous period of circadian rhythm with the external light/dark cycle provides a fitness advantage [19]. For example, *Arabidopsis* grown under a light/dark cycle that matches its circadian rhythm exhibits enhanced leaf chlorophyll content, net photosynthetic rate, plant growth, and better survival [19]. In our study, F_v_/F_m_ values ranged from 0.80 to 0.84 (Figure 4a,d), indicating the photosynthetic apparatus were not stressed under any of the light/dark cycles tested. However, domesticated cultivars (cv. ‘Frillice’ and ‘Crunchy’) exhibited different adaptive responses influenced by light/dark cycles, partly through changes in morphological characteristics (Figure 1 and Figure 2).

Under a light/dark cycle period of 24 h, a daily light integral of 14.4 mol m^−2^ d^−1^ combined with photoperiod of 16 h d^−1^ is recommended as the suitable light regime for commercial hydroponic lettuce production to achieve high yield and resource use efficiencies in the LED plant factory [21,26,27]. Following transplantation, a 21-day cultivation period is reasonable in the plant factory for hydroponic lettuce production to prevent tipburn occurrence [28]. In this study, the influences of light/dark cycle on lettuce growth were compared on the basis of the abovementioned light environment parameters. Our results show that the daily course of shoot fresh weight in both Frillice and Crunchy could be divided into slow and rapid growth stages over the 21 days after transplanting (Figure S1). Although differences in cultivars resulted in variations in growth curves, the “S” shape was consistent with previous reports [29,30].

It has been reported that shoot fresh or dry weight was higher under light/dark cycles of 16/8 and 8/4 compared with the 12/6 light/dark cycle in romaine lettuce [11]. In contrast, our findings show the opposite results—the light/dark cycle of 12/6 improved shoot fresh and dry weights on days 9 and 21 after transplanting in Frillice compared with the 16/8 light/dark cycle (Figure 5a,b,d,e). The increase was attributed to the larger leaf area, leaf number, and net photosynthetic rate (Figure 2 and Figure 3). Similarly, Crunchy exhibited increased shoot fresh and dry weights under light/dark cycles of 12/6 and 8/4 on day 9 after transplanting compared with the 16/8 light/dark cycle (Figure 5a,b), due to the larger leaf area and leaf number. However, under light/dark cycles of 16/8, Crunchy achieved faster growth during the rapid growth stage (Figure 5d,e), which was associated with a high growth rate (Figure 5f) caused by a high leaf number and large leaf area (Figure 2c,d), as well as a high net photosynthetic rate (Figure 3d), resulting in greater biomass accumulation (Figure 5e). The differences in leaf area may be attributed to cell cycles, which are regulated by the pseudo-response regulator TOC1 (TIMING OF CAB EXPRESSION1), a key component of the circadian system. TOC1 represses CELL DIVISION CONTROL 6, a DNA replication factor, to control the speed of cell cycles [31]. The interplay between circadian clock and cell cycle could influence leaf area and hypocotyl length [31]. Another possible explanation for the differences in shoot weight among light regimes was that light/dark cycles with appropriate intervals might help balance the positive regulation induced by light and darkness [32,33]. Moreover, the lowest shoot dry weight under a light/dark cycle of 4/2 in Crunchy could be explained by the excessive degree of reduction in light/dark cycle period, forcing leaves into frequent photosynthetic induction from darkness to light. This process results in a lagged photosynthetic rate as light intensity rapidly increases due to the activation of enzyme activity and stomatal opening, leading to less CO_2_ assimilation [34]. Morphological and physiological assessments suggest that the period of circadian rhythm was approximately 18 h for Frillice and 18–24 h for Crunchy. Higashi et al. (2014) reported that LED illumination shortened the circadian rhythms in lettuce cultivars (cv. ‘Cisco’, ‘Cos’, and ‘Greenwave’) from 19.9 h under monochromatic red light (0% blue) to 23.6 h under monochromatic blue light (100% blue) [35]. However, maximum lettuce growth did not occur in circadian rhythm periods of less than 24 h in Cos and Greenwave under a mixture of red and blue light [18]. These distinct results are likely due to genetic mutations resulting from selective breeding.

### 4.2. Light/Dark Cycles Can Regulate Water Use Efficiency by Altering Stomatal Opening

Water use efficiency could be manipulated by stomatal opening through altering stomatal density and size. Components of the circadian oscillator directly regulate the genes involved in stomatal function and stomatal aperture to influence water use efficiency [36]. The light/dark cycle could activate the abscisic acid signaling pathway by altering the expression of *LsROPGEF1*, resulting in stomatal closing [11,37]. In this study, stomatal conductance under a light/dark cycle of 16/8 in both Frillice and Crunchy decreased more significantly from the slow to rapid growth stage compared with other light/dark cycles (Figure 3b,e), resulting in higher water use efficiency (Figure 3f). These results indicate that light/dark cycles could regulate water use efficiency by altering CO_2_ assimilation and water loss, which were influenced by stomatal opening.

### 4.3. Optimizing Light/Dark Cycle Could Enhance Productivity and Reduce Energy Consumption

Despite the recent advancements in the luminous efficacy of LED lamps, lighting still accounts for 59% of total energy consumption in the plant factory [38]. The high operational costs have hindered the expansion of plant factories. To assess operational efficiency, researchers have proposed three key indicators—photon yield, light energy use efficiency, and electric energy use efficiency [20,21]. Under the same level of emitted photons, radiation, and electric energy consumption by LED lamps across different light/dark cycles, the highest photon yield, light energy use efficiency, and electric energy use efficiency were observed under a 12/6 light/dark cycle for Frillice and 12/6 or 16/8 light/dark cycle for Crunchy (Figure 6). These results are consistent with the shoot fresh or dry weight measurements (Figure 5e). This demonstrates that lettuce productivity could be significantly improved by optimizing the light regime while maintaining the same hardware platform. The findings provide scientific support for commercial lettuce production in energy-saving LED plant factories, contributing to its scale popularization. Our results show that, even under a fixed light/dark ratio, the growth responses of two lettuce cultivars to the light/dark cycle period were distinct. It could be speculated that the interactive effect of light/dark cycle ratio and light/dark cycle period on lettuce growth and its regulation mechanism might be complex. It still needs to be further researched and explained.

## 5. Conclusions

Different light/dark cycles significantly impacted morphology, photosynthetic performance, and energy use efficiency in lettuce cultivars. The responses to light/dark cycles varied depending on the cultivar. In a plant factory equipped with LED lamps, a 12/6 light/dark cycle was recommended to achieve high biomass accumulation and optimal LUE and EUE for Frillice. In contrast, a 16/8 light/dark cycle was more suitable for maximizing productivity and resource (light, electricity, and water) use efficiencies in Crunchy. These findings enhance our understanding of the cultivar-specific circadian rhythm in hydroponic lettuce at different growth stages, and provide valuable insights for optimizing the light regime to improve the productivity of hydroponic lettuce in an LED plant factory.

## Figures and Tables

**Table 1:** Combination of lettuce cultivar and light/dark cycle.

Cultivar	Treatment	Light Time (h)	Dark Time (h)	Total Light/Dark Cycles
Frillice	L_16_D_8_	16	8	21
	L_12_D_6_	12	6	28
	L_8_D_4_	8	4	42
	L_4_D_2_	4	2	84
Crunchy	L_16_D_8_	16	8	21
	L_12_D_6_	12	6	28
	L_8_D_4_	8	4	42
	L_4_D_2_	4	2	84

## Data Availability

Data will be made available on request.

## Supplementary Materials

The following supporting information can be downloaded at: https://www.mdpi.com/article/10.3390/biology14050571/s1, Figure S1: Time course of shoot fresh weight of hydroponic lettuces (cv. Frillice and Crunchy) after sowing.
