# Supplementary material for: Light/Dark Cycle Lighting Influences Growth and Energy Use Efficiency of Hydroponic Lettuces in an LED Plant Factory
Source: Biology (Basel). 2025 May 20;14(5):571. doi: 10.3390/biology14050571 (PMC12108944; doi:10.3390/biology14050571)
Supplement: Supplementary file 1 [file biology-14-00571-s001.zip › biology-3570046-supplementary.pdf]

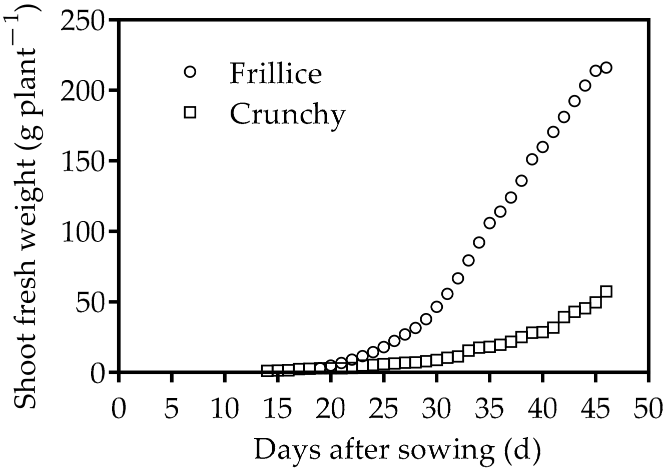

Figure S1. Time course of shoot fresh weight of hydroponic lettuces (cv. Frillice and Crunchy) after sowing.
